# A Non-Interventional Naturalistic Study of the Prescription Patterns of Antipsychotics in Patients with Schizophrenia from the Spanish Province of Tarragona

**DOI:** 10.1371/journal.pone.0139403

**Published:** 2015-10-01

**Authors:** Ana M. Gaviria, José G. Franco, Víctor Aguado, Guillem Rico, Javier Labad, Joan de Pablo, Elisabet Vilella

**Affiliations:** 1 Hospital Universitari Institut Pere Mata, Universitat Rovira i Virgili, CIBERSAM, IISPV Reus, Spain; 2 Hospital Universitari Institut Pere Mata, Reus, Spain; Benito Menni Complejo Asistencial en Salud Mental, SPAIN

## Abstract

**Background:**

The analysis of prescribing patterns in entire catchment areas contributes to global mapping of the use of antipsychotics and may improve treatment outcomes.

**Objective:**

To determine the pattern of long-term antipsychotic prescription in outpatients with schizophrenia in the province of Tarragona (Catalonia-Spain).

**Methods:**

A naturalistic, observational, retrospective, non-interventional study based on the analysis of registries of computerized medical records from an anonymized database of 1,765 patients with schizophrenia treated between 2011 and 2013.

**Results:**

The most used antipsychotic was risperidone, identified in 463 (26.3%) patients, followed by olanzapine in 249 (14.1%), paliperidone in 225 (12.7%), zuclopenthixol in 201 (11.4%), quetiapine in 141 (8%), aripiprazole in 100 (5.7%), and clozapine in 100 (5.7%). Almost 8 out of 10 patients (79.3%) were treated with atypical or second-generation antipsychotics. Long-acting injectable (LAI) formulations were used in 44.8% of patients. Antipsychotics were generally prescribed in their recommended doses, with clozapine, ziprasidone, LAI paliperidone, and LAI risperidone being prescribed at the higher end of their therapeutic ranges. Almost 7 out of 10 patients (69.6%) were on antipsychotic polypharmacy, and 81.4% were on psychiatric medications aside from antipsychotics. Being prescribed quetiapine (OR 14.24, 95% CI 4.94–40.97), LAI (OR 9.99, 95% CI 6.45–15.45), psychiatric co-medications (OR 4.25, 95% CI 2.72–6.64), and paliperidone (OR 3.13, 95% CI 1.23–7.92) were all associated with an increased likelihood of polypharmacy. Being prescribed risperidone (OR 0.54, 95% CI 0.35–0.83) and older age (OR 0.98, 95% CI 0.97–0.99) were related to a low polypharmacy probability.

**Conclusions:**

Polypharmacy is the most common pattern of antipsychotic use in this region of Spain. Use of atypical antipsychotics is extensive. Most patients receive psychiatric co-medications such as anxiolytics or antidepressants. Polypharmacy is associated with the use of quetiapine or paliperidone, use of a LAI, younger age, and psychiatric co-medication.

## Introduction

Antipsychotics are the mainstay treatment for schizophrenia and are used both to control acute relapses of symptoms and in maintenance therapy to reduce the risk of relapse [1,2]. They are classified into two main groups according to their chemical characteristics, effects on psychotic symptoms and adverse effect profiles: first-generation antipsychotics, or typical antipsychotics (TAP), and second-generation antipsychotics, or atypical antipsychotics (AAP) [3].

TAP are effective in alleviating positive symptoms but often cause unpleasant extrapyramidal effects that lead to poor treatment adherence. In addition to controlling positive symptoms, AAP might be better than TAP because they may have a relevant effect on negative and mood symptoms [4–6]. AAP are not a homogeneous class of drugs, and differences among them may influence clinicians when deciding to use one of them. A meta-analysis by Leucht *et al*. [7], which included 212 trials with 43,049 participants, concluded that AAP differ substantially in side effects (including metabolic and extrapyramidal), and also found small differences in efficacy among them. Some AAP (clozapine, amisulpride, olanzapine, and risperidone) were found to have higher efficacy than the TAP they were compared against in the meta-analysis (haloperidol and chlorpromazine). Other AAP, such as, aripiprazole, paliperidone, or ziprasidone had similar efficacy to TAP [7]. Clozapine is the most effective atypical antipsychotic and is useful in the treatment of resistant schizophrenia, but its use is limited due to the risk of agranulocytosis [4,6,7].

Despite the superiority of some AAP and the recommendation of clinical practice guidelines for treating schizophrenia with monotherapy [8–11], polypharmacy is a widespread practice around the world [12]. The rates of antipsychotic polypharmacy have been described as ranging from 4.1% to 57.7% [13–15]. Prescription patterns may vary from one country to another because they are determined by health care models, as well as because of cost and availability [12]. For example, a report of antipsychotic use patterns from Belgium showed that most patients were treated with AAP monotherapy [16], whereas a study from Germany found that only 10.5% of patients were treated with a single antipsychotic [17].

In Spain, the use of antipsychotics has increased remarkably in recent decades, going from 3 Defined Daily Doses [DDD]/1000 inhabitants/day in 1992 to over 8 DDD/1000 inhabitants/day in 2006, coinciding with the commercialization of diverse AAP [18]. Bernardo *et al*. [19] examined some patterns of antipsychotic prescriptions using the official medical prescription registry for the region of Barcelona (Catalonia, Spain). They found a 66.7% rate of antipsychotic monotherapy, with risperidone and olanzapine as the most frequently prescribed drugs, and a low frequency of clozapine use (2.3%). Unfortunately, their study did not record diagnostic categories, and so there is no way to know whether the antipsychotics were prescribed for schizophrenia, bipolar disorder or other psychiatric and neuropsychiatric conditions.

There are no published data on the prescription patterns for antipsychotics in all schizophrenia patients located in a specific catchment area from Spain. This is partially due to the lack of electronically recorded prescription data until relatively recently. In order to contribute to the global mapping of the use of antipsychotics in real-life settings, we describe the prescription patterns of antipsychotics in outpatients in the public mental health care network of services in the province of Tarragona (Catalonia, Spain). The frequency of the prescription of each medication, dosage, long-term antipsychotic polypharmacy, and the use of other psychiatric treatments are described.

## Methodology

### Design

A non-interventional, naturalistic, retrospective study was conducted based on registries of computerized medical records of all patients who were diagnosed with schizophrenia and treated in outpatient facilities in the public network of mental health in the province of Tarragona between 2011 and 2013. In accordance with Spanish law on the protection of personal data, the medical records were de-identified and anonymized prior to examination; all data management and analyses were carried out by personnel employed by our institution. The study was approved by the local Clinical Research Ethics Committee (Hospital Universitari de Sant Joan, Reus, Spain) and by the Clinical Research Committee of the Hospital Universitari Institut Pere Mata.

### Characteristics of the database

The data analyzed in this study were gathered from computerized or e-medical records registered in the electronic medical record (Ekon Salus System™). This application was used to manage data from all centers in the network. This system incorporates health management software with a post-relational database (InterSystems Caché). The e-medical record is specific to mental health services and has been developed by specialized medical personnel, as well as by experts in design and computer programming. It uses standardized classifications, such as the International Classification of Diseases, tenth edition (ICD-10), and the Anatomical Therapeutic Chemical Classification (ATC).

Extraction, transformation, and loading processes were created to acquire data from the production database and to store them in a Data Mart. Variables were located, evaluated, transformed, and standardized. To ensure data consistency and uniqueness, the team developed several quality systems, such as record duplication and detection and removal of inconsistencies and codification errors.

Data anonymization (in terms of patients, health care providers, and centers) was performed by the research team before data manipulation and analysis. Data were obtained via Structured Query Language queries on the database of medical records housed in our corporate network of mental health service providers.

The main operations carried out to deliver a consolidated and reliable data source were as follows: a) data location; b) data extraction; c) data transformation and standardization; d) data quality filtering; e) data anonymization; and f) construction of analytical tables.

### Study population

Tarragona is a province located in the south of the Region of Catalonia. The public mental health care network for Tarragona’s population is wholly served by the facilities of the Group Pere Mata and encompasses all services, including ambulatory mental health centers, emergency and hospital care, psychosocial rehabilitation, case management, and other specific facilities. All medical records are currently available in a computerized database.

The mean number of inhabitants of Tarragona during the study period was 782,766. Among them, 15,051 active patients were identified in the network of outpatient mental health services. All patients with a diagnosis of schizophrenia according to ICD-10 (F20.xx), over 18 years of age, and with at least one contact with a mental health center from the province between 2011 and 2013 were included. The prevalence of patients with schizophrenia treated in the outpatient network during the study period was 2.27/1,000. A total of 1,765 (11.7% of the active patients) met the selection criteria for this study. All records concerning antipsychotics and all other psychiatric medications that had been prescribed for at least three months were collected.

### Period of study and monitoring

The study period was from January 1, 2011, to December 31, 2013. Data were monitored until March 31, 2014 in order to ensure that each study patient had received active psychiatric medication for at least three months.

### Study variables

Demographic and clinical data, including schizophrenia subtype and psychiatric co-morbidities, were recorded. Patients were classified as suffering a severe mental disorder (SMD) when they met criteria for severity (i.e., proneness to deterioration, and marked alteration of personal, social, and family relationships as assessed by their psychiatrist) and persistence for more than two years.

All psychiatric drugs that were prescribed during the time period were recorded. Antipsychotics were identified based on the drugs within the code N05A, according to the ATC classification. A total of 25 drugs were identified and were classified as TAP or AAP, according to the consensus on the classification of antipsychotics [3].

The dose and route of administration were recorded for each drug, differentiating between oral (mg/day) and long-acting injection or LAI (mg/injection) prescriptions. The mean dose for oral treatment during the observation period was calculated for each patient by dividing the sum of the prescribed daily dose by the number of prescription days. Mean LAI doses were calculated by dividing the sum of mg/injection by the number of times that the patient was injected. Safety data sheets from the Spanish Medicines Agency were used for reference maintenance doses and ranges [20].

Patients were divided into two groups according to their long-term antipsychotic prescription pattern: the long-term polypharmacy group or the monotherapy group. Antipsychotic polypharmacy was defined as the identification of two or more antipsychotics that had been prescribed simultaneously for more than three consecutive months. The antipsychotic that was prescribed continuously for the longest time was identified as the principal drug, and its pattern of prescription was reported.

Concomitant prescriptions of other psychiatric drugs were recorded and coded according to the ATC classification as antiepileptic (N03A), anticholinergic (N04A), anxiolytic (N05B), hypnotic and sedative (N05C), or antidepressant (N06A).

### Statistical analysis

The analyses were performed using SPSS software Version 17.0 for Windows. Statistical significance of the two-tailed test was set at p<0.05. The normality of distribution of continuous variables was assessed with the Kolmogorov-Smirnov test.

Measures of central tendency ± standard deviation are shown for quantitative variables. Frequencies and percentages were used for qualitative variables.

Student's t-test or ANOVA were used to compare quantitative variables in the case of normal distributions; otherwise, the Mann-Whitney U or the Kruskal-Wallis test was used. The chi-squared test was used when contrasting categorical variables.

A backward stepwise logistic regression analysis using the likelihood ratio (LR) method and p<0.05 for entrance and p>0.10 for exclusion was performed to explore the potential association of antipsychotic polypharmacy with the five principal antipsychotics used most often, use of clozapine, LAI use, and other variables in the study. Hosmer and Lemesshow goodness of fit statistics and other literature recommendations were followed for logistic model construction [21]. Variables included in step 1 were: gender, age, marital status, schizophrenia subtype, SMD, psychiatric co-medication, psychiatric co-morbidity, consumption of substances, duration of disease; principal antipsychotic used (risperidone, olanzapine, paliperidone, zuclopenthixol, or quetiapine as dichotomous variables), clozapine use, and the use of LAI antipsychotics.

## Results

Five hundred and forty-eight (31%) of the 1,765 schizophrenia patients were female, and 1,217 (69%) were male. The mean age was 43.60±13.60 years.

The most frequent schizophrenia subtype was paranoid (73.3%), and the mean duration of the disorder was 13.90±7.10 years. More than half of the patients (64.8%) were classified as SMD cases. In addition, 40% of the patients had registered a psychiatric co-morbidity, and 27.7%, a substance abuse-related disorder (18.6% for tobacco and 9.1% for alcohol, THC, and cocaine combined); 1.1% had registered an affective disorder, and 1%, an anxiety disorder.

### Prescription patterns of the use of antipsychotics

Five hundred and five patients (28.6%) were on treatment with antipsychotic monotherapy, whereas 1,229 (69.6%) were on two or more antipsychotics (polypharmacy). Thirty-one patients (1.8%) were not taking antipsychotics. Table 1 shows the characteristics of patients according to their antipsychotic prescription pattern. The mean number of antipsychotics used was 2.42±1.47. A total of 532 (30.1%) patients had two antipsychotic prescriptions, 348 (19.1%) had three, and 179 (10.1%) had four. Univariate analysis showed that patients on antipsychotic polypharmacy were younger, used more substances, had a higher prevalence of psychiatric co-morbidity, and were less likely to be on AAP as the principal treatment.

**Table 1:** Demographic and clinical characteristics and patterns of antipsychotic use in 1,765 outpatients with schizophrenia. Values are frequencies (%) or means ± standard deviations.

Variable	Without AP (n = 31)	Monotherapy (n = 505)	Polypharmacy (n = 1,229)
**Gender**			
Male	19 (61.3)	330 (65.3)	868 (70.6)
Female	12 (38.7)	175 (34.7)	361 (29.4)
**Age**	45.58±13.04	45.82±13.80	**42.64±13.44**
Marital status			
Single	23 (74.2)	327 (64.8)	861 (70.1)
Married / Domestic Partner	6 (19.4)	74 (14.7)	154 (12.5)
Separated / Divorced	1 (3.2)	32 (6.3)	63 (5.1)
Widowed	-	8 (1.6)	13 (1.1)
Unknown/Not reported	1 (3.2)	64 (12.7)	138 (11.2)
**Schizophrenia subtype**			
F20.0 Paranoid	21 (67.7)	343 (67.9)	929 (75.6)
F20.1 Hebephrenic	-	13 (2.6)	33 (2.7)
F20.2 Catatonic	-	2 (0.4)	2 (0.2)
F20.3 Undifferentiated	4 (12.9)	31 (6.1)	64 (5.2)
F20.4 Post-schizophrenic depression	-	-	1 (0.1)
F20.5 Residual	3 (9.7)	66 (13.1)	109 (8.9)
F20.6 Simple	-	21 (4.2)	40 (3.3)
F20.8 Other	1 (3.2)	2 (0.4)	11 (0.9)
F20.9 Unspecified	2 (6.5)	27 (5.3)	40 (3.3)
**SMD**	17 (54.8)	312 (61.8)	815 (66.3)
**Principal Antipsychotic**			
Typical	-	72 (14.3)	**263 (21.4)**
Atypical	-	**433 (85.7)**	966 (78.6)
**Psychiatric co-medication**	12 (38.7)	340 (67.3)	**1,085 (88.3)**
**Psychiatric co-morbidity**	6 (19.4)	157 (31.1)	**543 (44.2)**
**Consumption of substances**	5 (41.7)	120 (54.5)	**446 (68.9)**
**Duration of disease (months)**	162.67±96.25	167.63±86.46	167.15±85.64

**Note**. SMD = Severe Mental Disorder. Values significantly different (according to Pearson’s Chi square or the Kruskal Wallis test) are in bold (p<0.01).

The frequencies and doses of each principal antipsychotic are presented in Table 2. LAI were prescribed in 790 (44.7%) patients, and 88.7% of the people on LAI were taking at least one other antipsychotic. AAP was the principal treatment for most of the patients (79.3%), with risperidone being the most prescribed antipsychotic (26.2%), followed by olanzapine (14.1%), paliperidone (12.7%), quetiapine (8%), aripiprazole and clozapine (both 5.7%). The most common TAP used was zuclopenthixol (11.4%), followed by fluphenazine (5.4%).

**Table 2:** Antipsychotic prescription patterns for 1,734 of 1,765 outpatients with schizophrenia. Recommended maintenance doses and corresponding ranges according to the Spanish Medicines Agency are shown if available. Data are for principal antipsychotic treatments. Oral antipsychotics are in italic (n = 944) and LAI are in bold (n = 790).

Antipsychotic	Recommended doses (range)	MONOTHERAPY (n = 505)		POLYPHARMACY (n = 1,229)	
		n (%)	Doses Mean±SD^a^	n (%)	Doses Mean±SD^a^
*Amisulpride*	(400–800)	13 (2.6)	503.26±306.92	62 (5)	567.78±293.36
*Aripiprazole*	15 (10–30)	42 (8.3)	14.03±6.91	58 (4.7)	16.70±7.51
*Asenapine*	10 (5–20)	-	-	4 (0.3)	12.70±4.88
*Chlorpromazine*	(75–150)	-	-	1 (0.1)	100.00
*Clozapine*	200 (200–450)	49 (9.7)	355.41±154.74	51 (4.1)	388.99±157.95
*Haloperidol*	3–9 (3–20)	17 (3.4)	7.99±6.02	2 (0.2)	8.50±4.95
*Levomepromazine*	75 (50–200)	2 (0.4)	125±35.35	5 (0.4)	22.73±6.43
*Olanzapine*	15 (5–20)	138 (27.3)	13.95±11.19	111 (9)	13.50±7.26
*Paliperidone*	6 (3–12)	6 (1.2)	6.58±3.33	20 (1.6)	6.72±2.24
*Perphenazine*	-	9 (1.8)	16.75±6.59	2 (0.2)	6.80±1.69
*Quetiapine*	600 (400–800)	14 (2.8)	418.65±299.25	127 (10.3)	320.37±222.72
*Risperidone*	(4–6)	109 (21.6)	4.12±2.35	54 (4.4)	5.31±5.01
*Sulpiride*	-	1 (0.2)	367.68	-	-
*Ziprasidone*	40 (40–160)	12 (2.4)	129.03±37.72	30 (2.4)	108.19±41.38
*Zuclopenthixol*	(20–50)	4 (0.8)	16.66±11.54	1 (0.1)	20.00
**Fluphenazine decanoate**	25 (12.5–50)	16 (3.2)	14.08±5.56	79 (6.4)	17.43±11.32
**Paliperidone palmitate**	75 (25–150)	6 (1.2)	93.91±9.16	193 (15.7)	111.56±39.91
**Risperidone microspheres**	25 (25–50)	44 (8.7)	42.64±17.98	256 (20.8)	46.11±18.40
**Zuclopenthixol decanoate**	200 (200–400)	23 (4.5)	147.41±80.84	173 (14.1)	170.75±84.28

***Note***. SD = Standard deviation; LAI = Long-acting injectable.

^a^ Doses are mg/day for oral antipsychotics and mg/injection for LAI.

Almost all of the antipsychotic doses were within the recommended maintenance ranges. Aripiprazole, asenapine, chlorpromazine, haloperidol, olanzapine, paliperidone (oral), and fluphenazine (LAI) were prescribed at their recommended maintenance doses. However, clozapine, ziprasidone, paliperidone (LAI), and risperidone (LAI) were prescribed at the upper end of their recommended ranges (Table 2).

Patients on AAP were younger than those on TAP (42.06±13.15 years vs. 49.85±13.77 years, t = 9.648, p<0.001) and had a higher prevalence of the schizophrenia paranoid subtype than did those on TAP (75.5% vs. 64.5%, χ^2^ = 52.261 df = 8 p<0.001). Those on TAP had a higher prevalence of SMD than did those on AAP (71.3% vs. 63.5%, χ^2^ = 7.357 df = 1 p<0.01), as well as a longer duration of disease (196.48±80.18 months for TAP vs.160.30±85.72 months for AAP, t = 7.317 df = 532.3 p<0.001). Anticholinergic use was higher in TAP patients than in AAP patients (49.9% vs. 27.4%, χ^2^ = 63.032 df = 1 p<0.001). S1 Table shows all of the demographic and clinical characteristics of the patients on TAP or AAP as the principal treatment.

Table 3 shows the frequencies for the principal combinations of antipsychotics. The most used antipsychotics in combination with others were olanzapine and quetiapine.

**Table 3:** Polypharmacy for the five most common principal antipsychotic treatments.

Principal antipsychotic	Add-on antipsychotic	n (% within group)
Risperidone 25.2% (n = 310)	Quetiapine	61 (19.7)
	Olanzapine	58 (18.7)
	Aripiprazole	52 (16.8)
	Clozapine	26 (8.4)
	Levomepromazine	23 (7.4)
	Other TAP low-potency	6 (1.9)
	Other TAP high-potency	24 (7.7)
	Other AAP low-potency	20 (6.4)
	Other AAP high-potency	40 (12.9)
Paliperidone 17.3% (n = 213)	Olanzapine	48 (22.5)
	Quetiapine	44 (20.7)
	Aripiprazole	29 (13.6)
	Amisulpride	14 (6.6)
	Ziprasidone	10 (4.7)
	Other TAP low-potency	9 (4.2)
	Other TAP high-potency	13 (6.1)
	Other AAP low-potency	8 (3.8)
	Other AAP high-potency	38 (17.8)
Zuclopenthixol 14.1% (n = 174)	Olanzapine	54 (31)
	Quetiapine	32 (18.4)
	Risperidone	18 (10.3)
	Aripiprazole	14 (8.1)
	Amisulpride	11 (6.3)
	Other TAP low-potency	11 (6.3)
	Other TAP high-potency	15 (8.6)
	Other AAP low-potency	9 (5.2)
	Other AAP high-potency	10 (5.7)
Quetiapine 10.3% (n = 127)	Olanzapine	31 (24.4)
	Aripiprazole	28 (22.1)
	Risperidone	20 (15.7)
	Haloperidol	11 (8.7)
	Paliperidone	9 (7.1)
	Other TAP low-potency	5 (3.9)
	Other TAP high-potency	1 (0.8)
	Other AAP low-potency	16 (12.6)
	Other AAP high-potency	6 (4.7)
Olanzapine 9% (n = 111)	Aripiprazole	37 (33.3)
	Risperidone	28 (25.2)
	Levomepromazine	11 (9.9)
	Haloperidol	9 (8.1)
	Clotiapine	5 (4.5)
	Other TAP low-potency	1 (0.9)
	Other TAP high-potency	6 (5.4)
	Other AAP low-potency	8 (7.2)
	Other AAP high-potency	6 (5.4)
Other TAP low-potency 0.5% (n = 6)	TAP high-potency	6 (100)
Other TAP high-potency 6.7% (n = 83)	TAP low-potency	7 (8.4)
	TAP high-potency	21 (25.3)
	AAP low-potency	43 (51.8)
	AAP high-potency	12 (14.5)
Other AAP low-potency 9.5% (n = 117)	TAP low-potency	6 (5.1)
	TAP high-potency	4 (3.4)
	AAP low-potency	60 (51.3)
	AAP high-potency	47 (40.2)
Other AAP high-potency 7.2% (n = 88)	TAP low-potency	4 (4.5)
	TAP high-potency	3 (3.4)
	AAP low-potency	46 (52.3)
	AAP high-potency	35 (39.8)

### Prescription patterns of psychiatric drugs other than antipsychotics

The majority of the patients included in the study, 1,437 (81.4%), were prescribed some type of psychiatric co-medication. The most prevalent concomitant medications were anxiolytics in 49% of the 1,437 cases, followed by antidepressants (36.9%), antiepileptics (32.9%), hypnotics / sedatives (32.5%), and anticholinergics (31.2%).

It is worth noting that according to the ATC, the antiepileptic group included derivatives of benzodiazepines, particularly clonazepam, which was the most used antiepileptic drug, prescribed to 17.8% of the patients.

**Fig 1:**
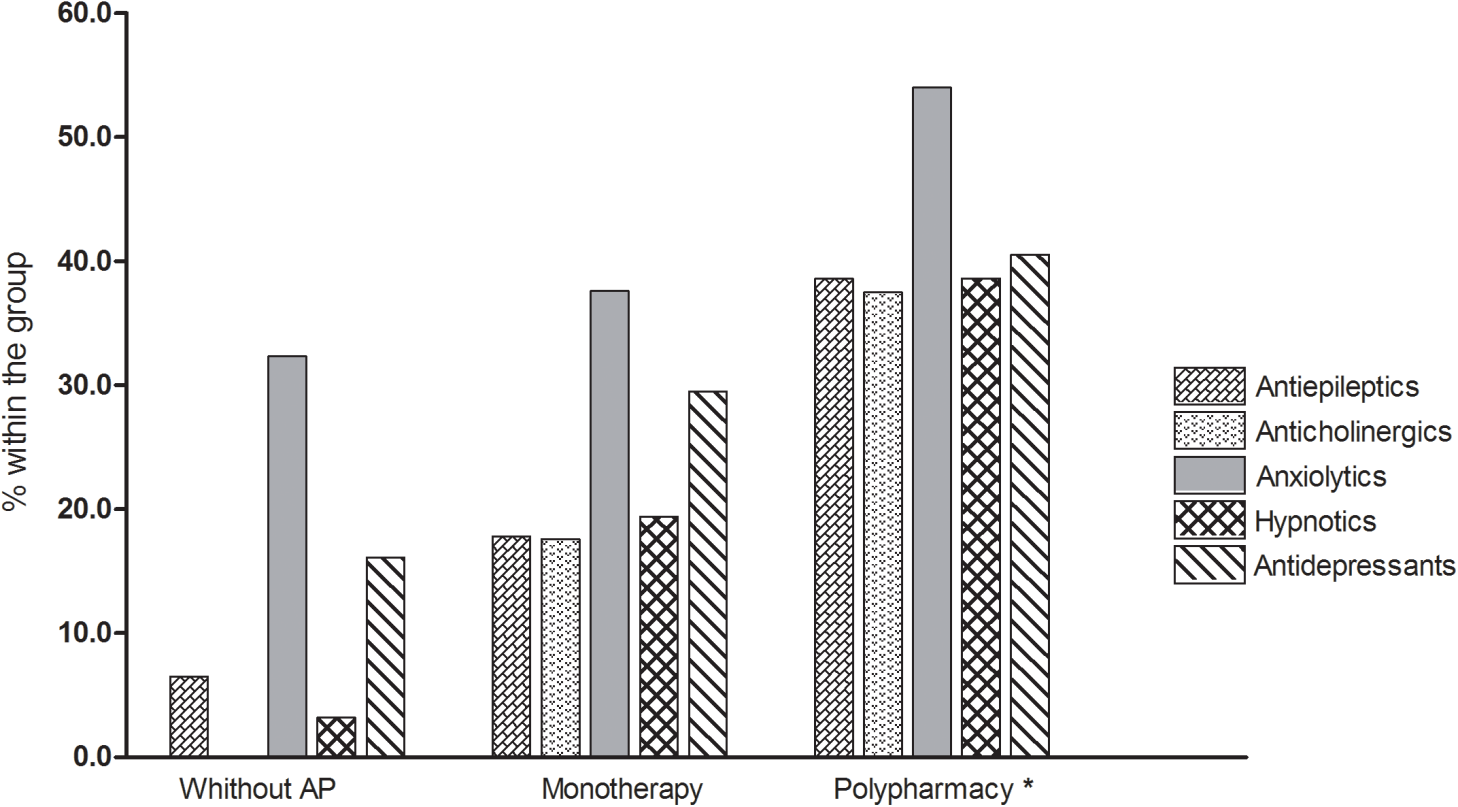
Comparison of the frequencies of psychiatric co-medications (other than antipsychotics) between the principal antipsychotic prescription patterns. An asterisk (*) indicates an observed frequency higher than expected in all χ^2^ comparisons, p<0.01.

More than half of the patients (61.5%) were treated with at least two antipsychotics and at least one psychiatric co-medication. The mean number of non-antipsychotic co-medications was 1.84±1.36. A total of 25.7% patients were taking one other psychiatric co-medication, 25.1% two, and 20% three medications. The frequency of psychiatric co-medication was significantly higher when this formed part of a pattern of antipsychotic polypharmacy (Fig 1).

The 1,399 patients who were on AAP as the principal treatment were more often on anxiolytics (50.9%), antidepressants (39.7%) and antiepileptic treatments (33.8%), (p<0.001 for all χ^2^). Half of the patients (50%) on TAP as the principal treatment were also receiving anticholinergic treatment (χ^2^ = 63.03, df = 1 p<0.001).

### Characteristics associated with antipsychotic polypharmacy

The results from the logistic regression analysis are shown in Table 4. Use of quetiapine as the principal treatment, use of an LAI, psychiatric medication co-pharmacy, and paliperidone as the principal treatment were all associated with a higher probability of being on polypharmacy. Use of risperidone as the principal treatment and older age were associated with a lower probability of being on polypharmacy.

**Table 4:** Variables associated with antipsychotic polypharmacy in patients with schizophrenia. Multivariate exploratory model using backward stepwise logistic regression.

	β (ET)	Wald	OR	95% CI
Constant	-0.565 (0.426)	1.763	0.568	
Male gender	0.332 (0.197)	2.844	1.394	0.948–2.052
Age	-0.017 (0.007)	6.014^*^	**0.983**	0.970–0.997
Psychiatric co-medication use	1.449 (0.227)	40.713^***^	4.259	2.729–6.646
Risperidone as principal treatment	-0.608 (0.216)	7.928^**^	**0.544**	0.357–0.831
Paliperidone as principal treatment	1.142 (0.474)	5.813^*^	3.132	1.238–7.925
Quetiapine as principal treatment	2.656 (0.539)	24.264^***^	14.240	4.949–40.974
LAI antipsychotic use	2.302 (0.223)	106.939^***^	9.991	6.459–15.455

***Notes***. Values in bold indicate an inverse relationship with polypharmacy (i.e., “protective factor” associated with monotherapy). *R* ^2^ = 0.377 (Nagelkerke). Model χ^2^_(8)_ = 7.996 p = 0.434. Significant using Wald’s test at

*p<0.05

**p<0.01

***p<0.001.

## Discussion

We describe patterns of prescribing antipsychotics in the schizophrenia patient population from a catchment area in a southern European province, considering the principal antipsychotic used and its dosage, long-term antipsychotic polymedication, and concomitant use of other psychiatric medications. An AAP was the principal treatment of choice in almost 8 of every 10 patients. Risperidone, olanzapine, and paliperidone were commonly prescribed, followed by the first TAP on the list (zuclopenthixol). Almost half of the patients were on a LAI. Antipsychotics were generally prescribed in the recommended therapeutic dose, though for clozapine, ziprasidone, paliperidone (LAI), and risperidone (LAI) it was at the upper end of their recommended ranges. Otherwise, 7 of every 10 patients were on antipsychotic polypharmacy, and the prescription of other psychiatric drugs was high, with almost half of the patients taking anxiolytics.

Our results were similar to those reported in outpatients with schizophrenia in New Zealand, for whom risperidone and olanzapine were the most frequently prescribed drugs [22]. In addition to being the most common oral antipsychotic, risperidone was the most common LAI in our study. Such findings are similar to those reported in other developed countries such as the United Kingdom [23] and the United States [24]. The preferential use of these two drugs may be a consequence of their entering the market earlier compared to other drugs such as quetiapine, as well as their diverse formulation possibilities (especially for risperidone which is available as an LAI and in various oral presentations). Administrative restrictions by the Health Care System on the use of the newer antipsychotics (aripiprazole and paliperidone) in our catchment area during the study period may also have influenced our specific pattern.

Though paliperidone was introduced in Spain as an LAI in late 2011 and there is a restriction on its use in Tarragona, it was the third most commonly prescribed drug among all antipsychotics and the second most common among LAIs, perhaps due to its more convenient monthly administration and its favorable metabolic profile compared with LAI risperidone [25]. The third most prescribed LAI in our area was a TAP (zuclopenthixol), which has been in use for many years.

Contrary to recommendations in the literature [8–11], the prescription frequency for antipsychotic monotherapy was low (less than 3 of every 10 patients), with the majority of patients being on antipsychotic polypharmacy and/or other psychiatric co-medication. According to the European College of Neuropsychopharmacology consensus on the advantages and disadvantages of combination treatment with antipsychotics, antipsychotic polypharmacy and the high use of other psychiatric medications, as found in this report, may be partially explained by the following four reasons [26]:

1. Partial response to monotherapy: Although the efficacy of combining antipsychotics for improving clinical response remains undemonstrated and clozapine is recommended for those who do not respond to two previous antipsychotics as monotherapy [8–11], it has been hypothesized that combining antipsychotics with strong D_2_ receptor binding properties with antipsychotics with weak D_2_ receptor binding may result in an optimal 70–80% receptor occupancy [27]. The combination of TAP (which improves positive symptoms) and AAP (which may improve negative symptoms) might also improve treatment efficacy [28]. An example of this type of combination in our study was the recurrent use of risperidone+quetiapine or zuclopenthixol+olanzapine.
2. Combination therapy may increase tolerability by reducing the adverse effects of higher doses. In addition to the effectiveness of aripiprazole monotherapy for schizophrenia [29,30], its mechanism of action (partial agonist/modulator at D2 receptors) makes it useful in normalizing serum prolactin levels when combined with other antipsychotics [31]. In our study population, aripiprazole was one of the five most commonly used antipsychotics as monotherapy, and it was used in many patients in conjunction with olanzapine, risperidone, paliperidone or quetiapine.
3. There may be possible benefits from a *de novo* combination. The combination of low-potency antipsychotics with others may improve insomnia or anxiety. Low-potency antipsychotics (quetiapine, levomepromazine) were frequently combined with high-potency antipsychotics in our study.
4. The presence of concurrent symptoms other than those of the primary disorder: There is a high prevalence of affective and anxiety symptoms in patients with schizophrenia, so patients may benefit from antidepressant and anxiolytic treatment. Antipsychotic side effects such as extrapyramidal symptoms may require treatment with anticholinergics. The frequency of co-morbid disorders in our study was very low, but use of concomitant anxiolytics, antidepressants, and antiepileptics (including clonazepam) was common, which may partially reflect the underassessment of concomitant disorders other than schizophrenia.

Despite all of these arguments supporting the use of polymedication, our results call attention to the long-lasting concerns about the discrepancy between guidelines and the real-world treatment of schizophrenia (the so called “dirty little secret” of psychiatry) [32,33]. Current guidelines basically recommend antipsychotic monotherapy for schizophrenia treatment with the combination of two or more antipsychotics only as a last resort and clozapine as the treatment of choice for cases that are resistant to individual therapies [8–11]. In contrast, like others [34–36], we found an extensive use of antipsychotic polypharmacy together with the relatively limited use of clozapine. Moreover, we are not the only ones to find use of the irrational quetiapine+olanzapine combination, where both drugs have a similar receptor profile, there is no evidence of additional efficacy, and there is a higher prevalence of adverse events [37,38]. We also found the use of three antipsychotics in almost 2 of every 10 patients.

The multivariate analysis of the relationship between diverse clinical characteristics and antipsychotic polypharmacy showed that a low-potency antipsychotic such as quetiapine, and LAI prescriptions had a high likelihood of being related to the prescription of multiple antipsychotics. A high-potency antipsychotic, such as risperidone, and younger age were protective against polypharmacy. It is noteworthy that this analysis based on clinical and pharmacological variables left approximately 60% of the variance unexplained. These unexplained reasons could be related to psychiatrists’ preferences or worries. For example, it is possible that psychiatrists are prone to continue long-term polypharmacy after trying to switch to avoid destabilizing a patient [39]. Psychiatrists may also prefer polypharmacy because of skepticism about the usefulness of algorithms (like those in guidelines) [33], or they may feel that polypharmacy is justified after non-response to or non-tolerance of clozapine, even though there is little evidence for the efficacy of this approach [40].

According to Correll *et al*. [40], those who use antipsychotic polypharmacy tend to be attending psychiatrists rather than residents, have been practicing for a longer time, see more patients per week, and have a preferred combination of antipsychotics. On the other hand, as suggested by Goren *et al* [35]., the reasons for clozapine underutilization could include a high prescribing burden (including registration requirements), significant side effects, and the requirement of frequent laboratory testing.

Future analyses of polypharmacy patterns might usefully include both clinical/pharmacological variables and the characterization of clinicians (including the evaluation of reasons for prescribing more than two long-term antipsychotics and the non-prescription of clozapine). Continuing medical education and training on prescribing antipsychotics, including LAI and clozapine, and the simplification of administrative requirements for prescribing this latter drug, could reverse the pattern of polypharmacy and low clozapine use. The impact of these interventions on clinical practice could be assessed by means of clinical trials.

In summary, contrary to the recommendation for monotherapy in the treatment of schizophrenia, antipsychotic polypharmacy is widespread and clozapine use is relatively scarce. The use of low-potency antipsychotics such as quetiapine is associated with polypharmacy, and patients who are on LAI also have a higher probability of being on polypharmacy. Antipsychotic dosage was concordant with Spanish safety data, though clozapine, ziprasidone, paliperidone (LAI), and risperidone (LAI) were prescribed at the upper end of their recommended ranges.

The strengths of this naturalistic study include its description of real-life practice and the coverage of a population of more than 1,700 adult patients with schizophrenia from a defined catchment area, without the use of exclusion criteria, making it a representative population of patients. However, even though we are the only providers of public mental health in our province, it is probable that we will have missed an unknown number of patients cared for by private practitioners or insurance companies. It is also probable that there are some patients who have not contacted a mental health professional. Other limitations of our study include the fact that the information was obtained from a secondary source and not from prospective structured evaluation of the patients and/or interview with their psychiatrists. The study design may have led to a less accurate registration of certain variables, such as secondary diagnosis, or to the loss of other information with clinical relevance, such as adherence to prescribed treatment. Finally, this study focused on long-term antipsychotic polypharmacy, whereas short-term polypharmacy or the switching patterns of prescriptions were not examined. Our team is working on a specific study about such prescription patterns for both oral and LAI treatments and their associated factors.

## Supporting Information

S1 Table Demographic and clinical characteristics of 1734 patients on typical or atypical antipsychotic treatment. Values are frequencies (%) or means ± standard deviation. Percentages are within group and comparisons between groups.
(DOCX)
