# Supplementary material for: A Non-Interventional Naturalistic Study of the Prescription Patterns of Antipsychotics in Patients with Schizophrenia from the Spanish Province of Tarragona
Source: PLoS One. 2015 Oct 1;10(10):e0139403. doi: 10.1371/journal.pone.0139403 (PMC4591292; doi:10.1371/journal.pone.0139403)
Supplement: S1 Table — Values are frequencies (%) or means ± standard deviation. Percentages are within group and comparisons between groups. (DOCX) [file pone.0139403.s001.docx]

**Supplemental S1 Table.** Demographic and clinical characteristics of 1,734 patients on typical or atypical antipsychotic treatment. Values are frequencies (%) or mean ± standard deviation. Percentages are within group and comparisons between groups.

| **Variable** | **TYPICAL AP**  **(n=335)** | **ATYPICAL AP**  **(n=1,399)** |
| --- | --- | --- |
| **Gender** |  |  |
| Male | 227 (67.8) | 971 (69.4) |
| Female | 108 (32.2) | 428 (30.6) |
| **Age** | **49.85±13.77** | 42.06±13.15 |
| **Marital status** |  |  |
| Single | 223 (66.6) | 965 (69) |
| Married / Domestic Partner | 58 (17.3) | 170 (12.2) |
| Separated / Divorced | 13 (3.9) | 82 (5.9) |
| Widowed | 4 (1.2) | 17 (1.2) |
| Unknown/Not reported | 37 (11) | 165 (11.8) |
| **Schizophrenia subtype** |  |  |
| F20.0 Paranoid | 216 (64.5) | **1,056 (75.5)** |
| F20.1 Hebephrenic | **14 (4.2)** | 33 (2.3) |
| F20.2 Catatonic | 2 (0.6) | 2 (0.1) |
| F20.3 Undifferentiated | 15 (4.5) | 80 (5.7) |
| F20.4 Post-schizophrenic depression | - | 1 (0.1) |
| F20.5 Residual | **64 (19.1)** | 111 (7.9) |
| F20.6 Simple | 16 (4.8) | 45 (3.2) |
| F20.8 Other | - | 13 (0.9) |
| F20.9 Unspecified | 8 (2.4) | 59 (4.2) |
| **SMD** | **239 (71.3)** | 888 (63.5) |
| **Psychiatric co-medication** | 278 (83) | 1147 (82) |
| Anticonvulsants | 91 (27.2) | **473 (33.8)** |
| Anticholinergics | **167 (49.9)** | 383 (27.4) |
| Anxiolytics | 142 (42.4) | **712 (50.9)** |
| Hypnotics / sedatives | 111 (33.1) | 462 (33) |
| Antidepressants | 91 (27.2) | **556 (39.7)** |
| **Psychiatric co-morbidity** | 132 (39.4) | 568 (40.6) |
| **Consumption of toxics** | 112 (65.1) | 454 (65.3) |
| **Duration of disease (months)** | **196.48±80.18** | 160.30±85.72 |

**Note**. SMD = Severe Mental Disorder. Values significantly higher (according to Pearson’s Chi square or Mann-Whitney U tests) among treatment columns are bolded (p<0.01).
